# Isolation and Preliminary Characterization of Proteinaceous Toxins with Insecticidal and Antibacterial Activities from Black Widow Spider (*L. tredecimguttatus*) Eggs

**DOI:** 10.3390/toxins7030886

**Published:** 2015-03-16

**Authors:** Qian Lei, Hai Yu, Xiaozhen Peng, Shuai Yan, Jirong Wang, Yizhong Yan, Xianchun Wang

**Affiliations:** Key Laboratory of Protein Chemistry and Developmental Biology of Ministry of Education, College of Life Sciences, Hunan Normal University, Changsha 410081, China; E-Mails: leiqian0607@163.com (Q.L.); Yuhai0326@163.com (H.Y.); peng112112@163.com (X.P.); acemalcolm@163.com (S.Y.); wangjimei79314@163.com (J.W.); yan_yizhong@hotmail.com (Y.Y.)

**Keywords:** black widow spider, egg, proteinaceous toxin, fractionation, characterization, *L. tredecimguttatus*, antibacterial, insecticidal

## Abstract

The eggs of black widow spider (*L. tredecimguttatus*) have been demonstrated to be rich in toxic proteinaceous components. The study on such active components is of theoretical and practical importance. In the present work, using a combination of multiple biochemical and biological strategies, we isolated and characterized the proteinaceous components from the aqueous extract of the black widow spider eggs. After gel filtration of the egg extract, the resulting main protein and peptide peaks were further fractionated by ion exchange chromatography and reversed-phase high performance liquid chromatography. Two proteinaceous components, named latroeggtoxin-III and latroeggtoxin-IV, respectively, were purified to homogeneity. Latroeggtoxin-III was demonstrated to have a molecular weight of about 36 kDa. Activity analysis indicated that latroeggtoxin-III exhibited neurotoxicity against cockroaches but had no obvious effect on mice, suggesting that it is an insect-specific toxin. Latroeggtoxin-IV, with a molecular weight of 3.6 kDa, was shown to be a broad-spectrum antibacterial peptide, showing inhibitory activity against all five species of bacteria tested, with the highest activity against *Staphylococcus aureus*. Finally, the implications of the proteinaceous toxins in egg protection and their potential applications were analyzed and discussed.

## 1. Introduction

The venom of spiders contains many different kinds of biologically active components including neurotoxins, many of which have been used as agent tools for neurobiological studies and as lead molecules in the development of new insecticides and pharmaceuticals [1,2,3]. To date, most of the related research has been focused on the toxins in the venom of spiders, and the study of the spider toxins from outside the venom glands is limited. Different from many other venomous animals such as snakes, scorpions and some other spider species that have toxins only in the venoms secreted by their venom glands, the black widow spider has toxins not only in its venom glands, but also throughout its body, including the legs and abdomen, and even in the eggs and the newborn spiders [4,5,6,7]. Study of the toxins in the materials other than the venom and investigation of the possible relationship between these toxins and those in the venom of the spider obviously have important theoretical and practical significance. Recently, we have been carrying out a systematic study on the toxicity of the black widow spider (*L. tredecimguttatus*) eggs and reported some related research results. Gel electrophoresis and mass spectrometry demonstrated that the eggs are rich in high-molecular-mass proteins and peptides below 5 kDa. The extract of the eggs has a strong toxicity towards mammals and insects. The mammal toxicity of the eggs is primarily due to the high-molecular-mass proteins in the eggs. This extract could completely block the neuromuscular transmission in mouse isolated phrenic nerve-hemidiaphragm preparations. Using the whole-cell patch-clamp technique, the egg extract was demonstrated to be able to inhibit the voltage-activated Na^+^, K^+^ and Ca^2+^ currents in rat DRG neurons. In addition, the extract displayed activities of multiple hydrolases [8]. Comparative proteomic analysis indicates that the protein composition of the eggs is more complex than that of venom and there are only a few similarities between the protein compositions of the two materials, suggesting that the eggs have their own distinct toxic mechanism [9]. By using gel filtration combined with ion-exchange chromatography as well as RP-HPLC, two active proteins were purified from the eggs, named Latroeggtoxin-I and Latroeggtoxin-II, respectively. They have been demonstrated to be novel neurotoxins purified from the eggs of black widow spiders [10,11].

Here, we report another part of the systematic work, in which the aqueous extract of the black widow spider eggs was fractionated and characterized. Two toxic proteinaceous components, named Latroeggtoxin-III and Latroeggtoxin-IV, respectively, were purified to homogeneity from the extract and then screened for their physiochemical and biological properties using multiple analytical techniques.

## 2. Results

### 2.1. Molecular Sieve Chromatography of Egg Extract

**Figure 1:**
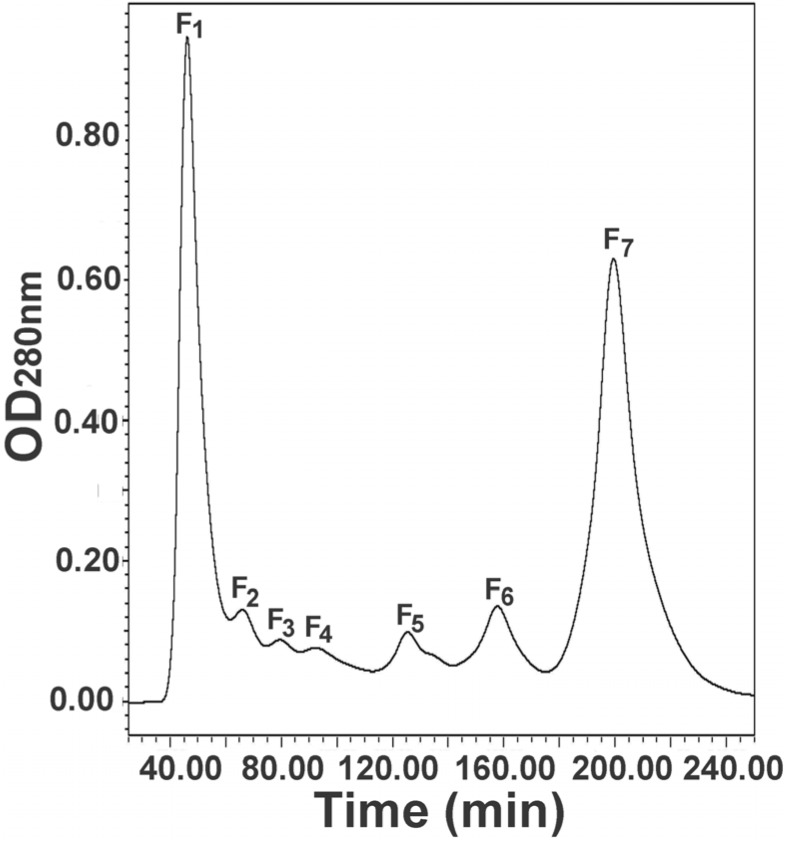
Molecular sieve chromatography of the egg extract of black widow spider. The egg extract was fractionated into seven fractions, named F_1_ to F_7_.

The egg extract was first fractionated with molecular sieve chromatography to separate the components based on their size. As a result, the components in the extract were fractionated into seven major fractions, marked with F_1_ to F_7_ (Figure 1). SDS-PAGE demonstrated that the first four fractions (F_1_ to F_4_) were primarily composed of high-molecular-weight proteins and the last three fractions (F_5_ to F_7_) consisted of mainly low-molecular-weight components (Figure S1 in Supplementary Figures) [10]. Of the resulting fractions, fractions F_1_ and F_7_ were the largest protein and peptide peaks, respectively, and were collected as the fractions for further separation.

### 2.2. Further Separation of Protein Fraction F_1_

**Figure 2:**
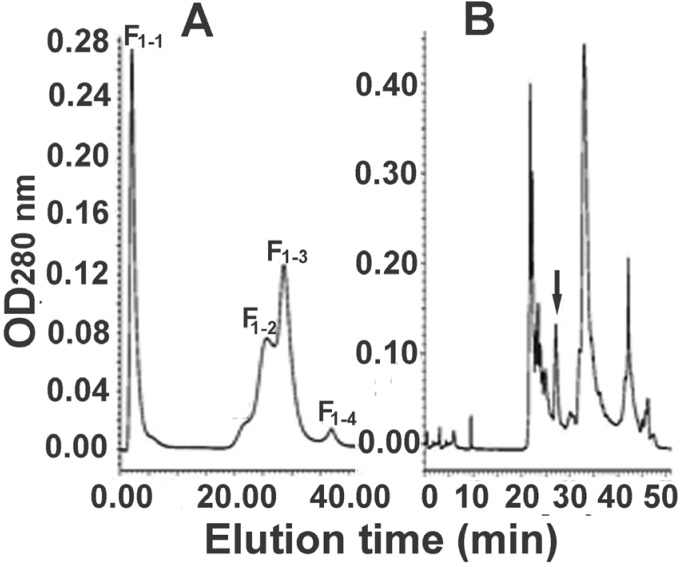
Further separation of the fraction F_1_ from molecular sieve chromatography. (**A**) Anion exchange chromatography of the fraction F_1_; F_1_ was the first fraction obtained from molecular sieve chromatography in Figure 1 and was further separated into four peaks, named F_1-1_ to F_1-4_; (**B**) RP-HPLC of the F_1-3_ from anion exchange chromatography of the fraction F_1_. The peak indicated with an arrow was Latroeggtoxin-III.

**Figure 3:**
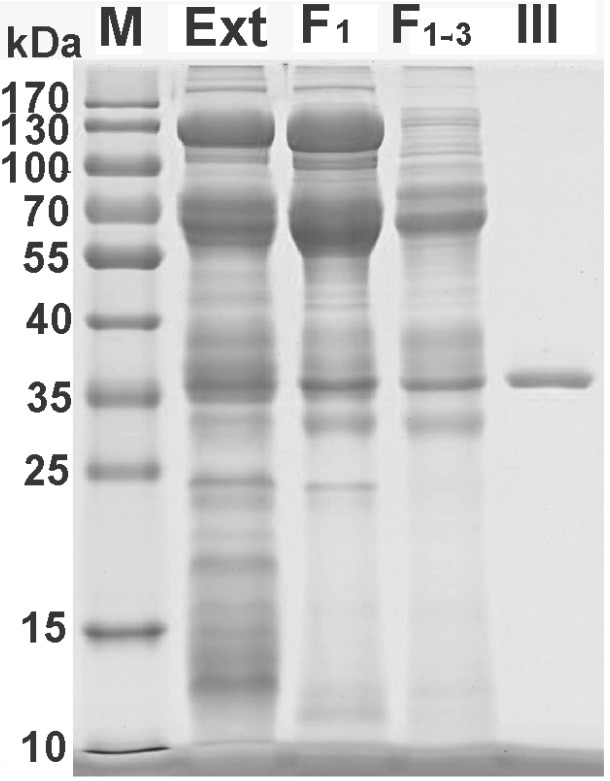
SDS-PAGE of egg extract and the samples resulting from each separation steps. M, molecular weight marker; Ext, egg extract; F_1_, fraction F_1_ from molecular sieve chromatography; F_1-3_, main peak resulting from the anion exchange chromatography of the fraction F_1_; III, Latroeggtoxin-III.

For further separation of the fraction F_1_, anion exchange and reversed-phase chromatographies were employed sequentially. A representative elution profile of anion exchange chromatography is shown in Figure 2A. From the figure it can be seen that the components in the fraction F_1_ were fractionated into four peaks (F_1-1_ to F_1-4_) and the complexity of the sample was further decreased. The main peak F_1-3_ was collected and was further separated with RP-HPLC. In view of the fact that the proteins to be isolated have a high molecular weight, we used a low-hydrophobicity C4 column in order to minimize the risk of protein denaturation. The representative RP-HPLC chromatogram is shown in Figure 2B. The peak indicated with an arrow was collected, which, together with egg extract and the partially purified samples, was analyzed by SDS-PAGE (Figure 3) to show purification efficiency in comparison. The results in the figure indicated that a combination of different chromatographic techniques such as molecular sieve filtration, ion-exchange and RP-HPLC could efficiently separate the proteins in the egg extract. During the purification processes the sample complexity was gradually decreased, and as a result a protein with a molecular weight of about 36 kDa was purified. The protein was named latroeggtoxin-III, because in previous work we had purified and characterized two different proteins (named Latroeggtoxin-I and Latroeggtoxin-II, respectively) from the black widow spider eggs [10,11].

### 2.3. Further Separation of Peptide Fraction F_7_

**Figure 4:**
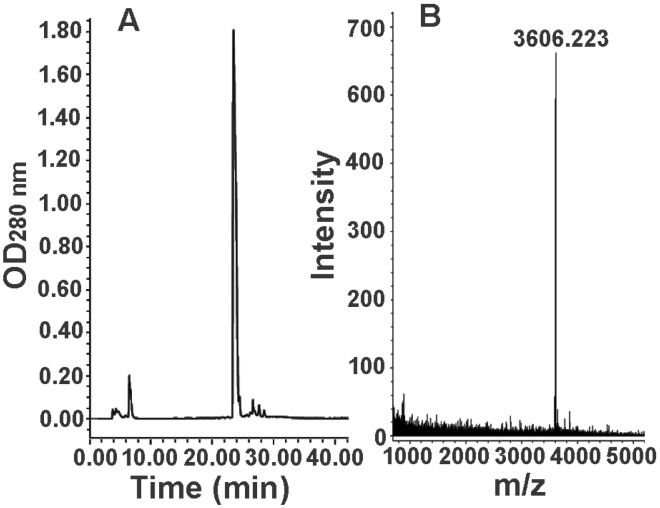
Further separation of fraction F_7_ from the molecular sieve chromatography and mass spectrometric analysis of the purified peptide. (**A**) RP-HPLC of the fraction F_7_; (**B**) MALDI-TOF mass spectrometric analysis of the purified peptide.

In view of the low-molecular-weight peptide composition of the fraction F_7_ from the molecular sieve chromatography, the further separation of the fraction F_7_ was carried out on a reversed-phase C18 column. The representative chromatogram is shown in Figure 4A, from which it can be found that the fraction F_7_ was separated into a major peak and several minor peaks. MALDI-TOF mass spectrometry indicated that the major peak contained a single component with a molecular weight (M + H^+^) of 3606.223 (Figure 4B), suggesting that a peptide had been purified in homogeneous form from the eggs of black widow spider. We named the peptide Latroeggtoxin-IV.

### 2.4. Bioactivity and N-terminal Sequence of Latroeggtoxin-III

To detect animal toxicity (if present) of Latroeggtoxin-III, the protein was injected intraperitoneally into mice and cockroaches (*P. americana*) and then their behaviors were observed. It was found that, at the experimental doses (up to 7.8 mg/kg body weight) used, the mice acted normally and did not display obvious poisoning symptoms, only being slightly more active than the control mice. In order to further probe into the animal toxicity of the Latroeggtoxin-III, isolated mouse phrenic nerve-diaphragm preparations were prepared and the effects of Latroeggtoxin-III on the nerve-muscle transmission in the preparations were investigated. The results indicated that Latroeggtoxin-III at a concentration of 50 µg/mL did not significantly alter the contraction amplitude of the diaphragm caused by electrically stimulating the phrenic nerve within 120 min (Figure S2 in Supplementary Figures). The above results suggested that mammalian toxicity of Latroeggtoxin-III, if present, is very weak.

**Figure 5:**
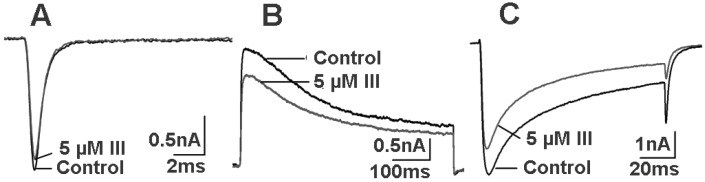
Effects of Latroeggtoxin-III on the sodium (**A**); potassium (**B**); and calcium (**C**) channel currents in cockroach dorsal unpaired median (DUM) neurons.

However, Latroeggtoxin-III had obvious toxic effects on *P. americana*. After being intraperitoneally injected with the protein at a dose of 10 μg/g body weight, the cockroaches displayed a series of obvious poisoning symptoms, including body turning, sluggishness and slow response, and finally died within 30 h. When the dose was increased, the survival time of the cockroaches was shortened. To further validate the neurotoxicity of Latroeggtoxin-III towards cockroaches, the whole-cell patch-clamp technique was employed to detect the effects of the protein on the ion channel currents in cockroach dorsal unpaired median (DUM) neurons. The results (Figure 5) indicated that Latroeggtoxin-III showed inhibitory actions on the ion channel currents particularly the potassium and calcium channel currents, which further validated the neurotoxicity toxicity of the Latroeggtoxin-III towards the insects.

The *N*-terminal sequence of Latroeggtoxin-III was identified by Edman degradation to be STKSSESLYLEALYIDKMTHEPVAD. When this sequence was utilized to perform Sequence query and homology analysis with Mascot and BLAST softwares against protein databases, respectively, no completely matched proteins were found. All the data together demonstrate that Latroeggtoxin-III is a kind of novel insect-specific proteinaceous toxin purified from the eggs of black widow spiders.

### 2.5. Bioactivity of Latroeggtoxin-IV

Toxicity experiments indicated that intraperitoneally injecting Latroeggtoxin-IV into mice and cockroaches *P. Americana* at doses of 3.32 mg/kg body weight and 20 µg/g body weight, respectively, led to no obvious poisoning symptoms of the animals. In addition, mouse phrenic nerve-hemidiaphragm preparation experiments (Figure S3 in Supplementary Figures) and whole-cell patch-clamp analyses with rat DRG neurons (Figure S4 in Supplementary Figures) indicated that the peptide had no or very weak neurotoxicity. These results demonstrated that Latroeggtoxin-IV is not a toxin for mice and cockroaches. However, it was found that the peptide exhibited a broad spectrum of antibacterial activity. In the present study, the Latroeggtoxin-IV was tested using the agar-disc diffusion assay for its antibacterial activity against two Gram-positive bacterial strains (*S. aureus* and *B. subtilis*) and three Gram-negative bacterial strains (*E. coli*, *S. typhimurium* and *P. aeruginosa*). The determined inhibition zone diameter (IZD) values are shown in Table 1. As shown in the table, Latroeggtoxin-IV (1.8 µg/disc) exhibited inhibitory activity against both of the Gram (+) and Gram (−) bacteria tested, producing inhibition zones varying from 9.46 ± 0.19 mm to 14.73 ± 0.96 mm. Among the sensitive strains, the largest inhibition zone was observed against *S. aureus* (14.73 ± 0.96 mm), followed by *S. typhimurium* (11.53 ± 0.16) and *B. subtilis* (11.40 ± 0.89 mm). Relatively, *P. aeruginosa* was least sensitive to Latroeggtoxin-IV. The inhibition zone of ampicillin (10 µg/disc) that was used as a positive control for bacteria ranged from 11.17 ± 0.41 to 13.49 ± 1.57 mm, which confirmed the authenticity of antibacterial activity measured under the present experimental conditions and could be used as a reference for roughly estimating the antibacterial potency of Latroeggtoxin-IV.

**Table 1:** Antibacterial activities of Latroeggtoxin-IV ^a^.

Strains	IZD ^b^	IZD ^c^
*S. aureus*	14.73 ± 0.96	11.17 ± 0.41
*S. typhimurium*	11.53 ± 0.16	12.58 ± 0.41
*B. subtilis*	11.40 ± 0.89	13.28 ± 0.44
*E. coli*	10.82 ± 2.00	13.49 ± 1.57
*P. aeruginosa*	9.46 ± 0.19	12.67 ± 0.33

^a^ Results are means of three different experiments; ^b^ Inhibition zone diameter of Latroeggtoxin-IV (1.8 µg/disc) in millimeters; ^c^ Inhibition zone diameter of ampicililin (10 µg/disc) that was used as a positive control.

## 3. Discussion

It has been demonstrated that the eggs of the black widow spider contain large amounts of proteinaceous components with different biological activities [4,5,8]. Our previous studies had purified and characterized two toxic proteins (Latroeggtoxin-I and -II) from the eggs [10,11]. In this study, we report the isolation and biological activity screening of two additional proteinaceous components, Latroeggtoxin-III and Latroeggtoxin-IV, from the spider eggs. By combining multiple biochemical techniques, these two components were separated to homogeneity, validated by gel electrophoresis and MALDI-TOF mass spectrometry, respectively. Toxicity analysis experiments suggested that, different from Latroeggtoxin-I and II, Latroeggtoxin-III and IV have no obvious mammalian toxicity and are cockroach-specific protein toxins and antibacterial peptides, respectively.

When we utilized the *N*-terminal sequence of Latroeggtoxin-III to perform a homology analysis using the protein BLAST program (Search protein database using a protein query), no completely matched proteins or sequences were found. However, when the sequence was used to search a transcriptome database from *Latrodectus Hesperus* (Search translated nucleotide database using a protein query), this sequence was found to match with a fragment of a 200-kDa predicted protein in the database. Furthermore, if the whole sequence of the predicted protein is used to search the nrNCBI protein database, proteins that are similar to vitellogenin were identified, suggesting that Latroeggtoxin-III might be a proteolytically-cleaved product of vitellogenin. Vitellogenin is known to be stored in the yolk and subject to cleavage to generate a host of products for the developing embryos. Vitellogenin and its proteolytically-cleaved product were previously known to form the yolk proteins, providing the energy reserves for developing embryos. However, there have been a series of experiments demonstrating that their roles extend beyond this nutrient function. For example, the honeybee vitellogenin has been demonstrated to be able to reduce oxidative stress [12]. The chicken egg yolk Pv, a vitellogenin-derived protein, was found to display an antibacterial effect against *Escherichia coli* [13]. Dreon *et al*. [14] purified a protein neurotoxin from the snail (*P. canaliculata*) eggs. To the best of our knowledge, Latroeggtoxin-III is the first toxic peptide that is purified from *L. tredecimguttatus* eggs and has high homology with vitellognin.

In order to further characterize Latroeggtoxin-IV, the peptide was subjected to sequencing by Edman degradation. Surprisingly, after the first Edman degradation cycle, it gave an obvious signal corresponding to Trp, and there were no other PTH-amino acid signals appearing in the following four consecutive cycles (Figure S5 in Supplementary Figures showing the chromatograms of the five cycles), suggesting the presence of a special structure, such as an intramolecular cycle, that prevented the sequencing from proceeding further. To further confirm the peptidic nature of the sample, we analyzed its amino acid composition and the results showed that the peptide contains most of the standard amino acids including Cys, Asp, Glu, Arg, Pro, Ser, Leu, *etc.* In addition, the UV absorption spectrum of the peptide displayed a strong characteristic absorption peak at 280 nm, which supported the conclusion that the peptide contains Trp residue(s). All of the results demonstrate that Latroeggtoxin-IV is a peptide with a structural peculiarity, the determination of which is in progress with a combination of different techniques including partial enzymolysis and tandem mass spectrometry.

Spider venoms are complex chemical mixtures that have evolved to kill or paralyze arthropod preys [15,16]. The polypeptide components are produced in a combinational fashion and tend to be the main constituent and active components of most spider venoms [1,15]. A notable exception is the venom of black widow spiders, which contain a high proportion of proteinaceous components with a high molecular weight and most of the toxic components are insect-specific. To date, the venom has been found to contain five insecticidal toxins, termed α-, β-, γ-, δ- and ε-latroinsectotoxins (LITs) [16,17,18]. The study on these components can not only give important clues as to the architecture and modes of action of the insect-specific toxins, but also explore their potential as insecticides and the tools to probe into neuroexocytosis, *etc*. When considering the use of LITs as potential insecticides, delivery of these large proteins can be realized based on the use of recombinant baculoviruses carrying the appropriate LIT genes. This strategy has effectively been tested with α-latroinsectotoxin [19]. Our present study demonstrates that the insecticidal proteins exist not only in the venom of black widow spiders, but also in their eggs. BLAST analysis utilizing the *N*-terminal sequence of the Latroeggtoxin-III indicated that the insecticidal protein in the eggs is different from those in the venom, which supported the conclusion that the eggs of black widow spider have their own distinct toxic mechanism [9]. The discovery of the novel insecticidal proteinaceous component from the eggs black widow spiders not only helps us to further understand the toxicity mechanism of the eggs, but also provides us with a new candidate for insecticidal agent development. Why the eggs of black widow spider, like the venom, have evolved to kill or paralyze arthropod preys is of interest. It was speculated that the existence of such components in the eggs could provide a certain protection for the eggs from some greedy arthropods, which was supported by the report of Russell *et al*. [20]. They demonstrated that *Latrodectus* egg poison had deleterious effects on the web-building activity of *Araneus diadematus*. The web-building activity of the spiders receiving 3–5 g/kg body weight was abnormal and one spider receiving 1 g/kg body weight died 6 h after feeding.

During the screening of potential bioactivities of Latroeggtoxin-IV, we found that the peptide toxin is a broad-spectrum antibacterial agent and shows antimicrobial activity against both Gram (+) and Gram (−) bacteria, with the highest activity on *Staphylococcus aureus*. Literature survey suggested that, although this is the first report on purification and characterization of an antimicrobial peptide from the eggs of black widow spiders, there are a patch of antimicrobial peptides having been isolated from the venoms of some other species of spiders. For example, Yan *et al*. [21] isolated two antibacterial peptides from venom of the wolf spider (*Lycosa carolinensis*), named lycotoxin I and II, both of which were predicted to have amphipathic α-helix character typical of antimicrobial pore-forming activity. Lycosin-I, a 24-residue cationic peptide from the venom of the spider *Lycosa singorensis*, was demonstrated to show rapid, selective and broad-spectrum antibacterial activity [22]. Kuhn-Nentwig *et al*. [23] isolated a new family of highly basic antimicrobial peptides (Cupiennin 1) from the venom of the spider *Cupiennius salei* (Ctenidae) and demonstrated that the cupiennins showed minimal inhibitory concentrations for bacteria in the submicromolar range. Their immediate biological effects and the structural properties indicate a membrane-destroying mode of action on prokaryotic as well as eukaryotic cells. It was speculated that those antimicrobial peptides may play a dual role in spider-prey interaction, functioning both in the prey capture strategy as well as to protect the spider from potentially infectious organisms arising from prey ingestion [21]. The adult female black widow spiders often suspend their egg sacs be from the ceiling deep in the retreat [24], which suggests that there must be specific mechanisms to protect the eggs from potentially pathogenic microorganisms. Thus, it is speculated that the peptide may play roles in protecting the eggs from some greedy animals and pathogenic microorganisms.

## 4. Experimental Section

### 4.1. Materials

Acetonitrile (ACN) and trifluoroacetic acid (TFA) were purchased from Sigma (St. Louis, MO, USA). Acrylamide, Bis, Tris, glycine and SDS-PAGE protein standards were from Fermentas (PageRuler; Burlington, ON, Canada). Ammonium persulfate, urea, agarose, glycerol, bromophenol blue and N, N, N', N'-tetramethylethylenediamine (TEMED) were from Amersham Pharmacia Biotech (Little Chalfont, UK). Molecular sieve gel Sephacryl™ S-200 was from GE Healthcare (Piscataway, NJ, USA). Yeast extract, tryptone and agar were from Shanghai Biological Engineering Co. Ltd of China.

### 4.2. Preparation of Egg Extract

The extract of black widow spider eggs was prepared using the methods previously described [4,10]. Briefly, the eggs were homogenized in a neutral PBS buffer of weak ionic strength or ddH_2_O with a mortar and pestle, and the resulting homogenate was centrifuged at 10,000 g for 10 min at 4 °C to separate supernatant and the pellet. The supernatant was collected and the pellet was repeatedly homogenized and extracted twice. All the collected supernatants were combined, lyophilized or appropriately concentrated in vacuum and stored at −20 °C for further analysis.

### 4.3. Fractionation of Egg Extract

The egg extract was fractionated on a molecular sieve chromatographic column (2.6 cm id × 60 cm long) packed with molecular sieve gel Sephacryl™ S-200 on a Waters™ 650 advanced protein purification system equipped with a model 485 detector. The column was first equilibrated with a dilute PBS buffer and then loaded with the extract sample, followed by elution with the same buffer solution at a flow rate of 2.5 mL/min. The optical density of eluate was monitored at 280 nm and the fractions were separately collected and appropriately concentrated. SDS-PAGE was employed to detect the molecular weight distribution of proteinaceous components in each fraction.

### 4.4. Detection of Molecular Weight Distribution of the Fractions

SDS-PAGE was used for analyzing the molecular eight distribution of each fraction and was performed according to the method of LaemmLi [25] on a 4.8% stacking gel and a 10% separation gel (1 mm thick). Aliquots of each fraction were separately dissolved in 30 μL of sample buffer (50 mM Tris-HCl, pH 6.8, 65 mM DTT, 0.5 mM phenylmethylsulfonyl fluoride (PMSF), 2% SDS, and a trace of bromophenol blue) and heated at 90 °C for 10 min. The sample solutions were centrifuged at 10,000× *g* for 15 min and the supernatants were loaded into the parallel sample wells in the gel. The SDS-PAGE was run at 25 mA on the stacking gel and at 50 mA on the separating gel. After complete of the electrophoresis, the separated proteins were visualized by Coomassie brilliant blue G-250 staining. A prestained protein ladder (PageRuler™ Fermentas, Burlington, ON, Canada) was used as standard molecular weight markers.

### 4.5. Further Isolation of Protein Fraction F_1_ and Peptide Fraction F_7_

The protein fraction F_1_ from gel filtration chromatography was further isolated with a TOYOPEARL DEAE-650M anion exchange column (TOSOH Co., Tokyo, Japan) (5 mm id × 10 cm long) on a Waters™ 650E Advanced Protein Purification System (Milford, MA, USA). After the column was sequentially washed with buffer A (50 mM Tris-HCl, 1.0 M NaCl, pH 8.5) and buffer B (50 mM Tris-HCl, pH 8.5), the sample was loaded and then eluted by gradually increasing the concentration of NaCl in the buffer B. The optical density of eluate was monitored at 280 nm using a Waters™ 486 tunable absorbance detector. The fraction of interest from the anion exchange chromatography was desalted and further purified using a C4 reversed-phase column (4.6 × 250 mm, Elite, Dalian, China) on a Waters HPLC system (Model Alliance 2690, Waters, Milford, MA, USA) with a 996-photodiode array detector. Mobile phase A was 0.05% TFA, and mobile phase B was ACN containing 0.05% TFA. After the sample was loaded, the column was eluted to remove the salts and further separate the absorbed proteins using a gradient elution as follows: 0–10 min, 100% A; 10–15 min, 0%–40% B; 15–35 min, 40%–50% B; 35–40 min, 50%–100% B, followed by 100% B for 10 min. The flow rate was 1.0 mL/min. Effluent absorbance was recorded at 280 nm. The resulting fractions were separately collected and lyophilized, followed by analysis with SDS-PAGE (Bio-Rad Co., Hercules, CA, USA).

The peptide fraction F_7_ from molecular sieve chromatography was desalted and further purified on the same Waters HPLC system (Model Alliance 2690, Waters, Milford, MA, USA) and under the same conditions except that a C18 reversed-phase column (4.6 mm× 250 mm, Elite, Dalian, China) was used. The sample purity and the molecular weight of the purified peptide were determined by matrix-assisted laser desorption/ionization- time of flight (MALDI-TOF) mass spectrometric analysis in a reflector mode (UltraFlex, Bruker Daltonics Ins., Billerica, MA, USA).

### 4.6. Animal Toxicity Detection

Aliquots of purified protein and peptide were intraperitoneally injected into mice and cockroaches *P. americana* and then the behavior of the animals was observed within the following 48 h in order to detect whether the components have mammal and/or insect toxicities. For insect toxicity detection, three cockroaches were used in an experiment. To each cockroach 10 µL of sample solution was injected between the fourth and fifth sternites of the cockroaches. The control cockroaches were injected with the same volume of physiological saline. The experiments were performed at least in triplicate. Then, their possible effects on neuromuscular transmission and on sodium, potassium and calcium ion channels in neurons were detected.

For detecting the potential effects of the purified samples on neuromuscular transmission, mouse isolated phrenic nerve-hemidiaphragm preparations were used and the experiments were performed according to method described previously [26]. Briefly, adult Kunming albino mice were killed by cervical dislocation immediately after anesthesia and the phrenic nerve-hemidiaphragm was dissected out and immersed in Tyrode’s solution contained in a small Plexiglas chamber, continuously bubbled with a mixture of 95% O_2_ and 5% CO_2_. The temperature of the Tyrode’s solution was maintained at 30–32 °C with a constant temperature circulating water bath device. Electrical stimulation was applied to the phrenic nerve with a suction electrode (supramaximal voltage, 2 ms duration, square wave) and the resulting twitch responses of diaphragm muscle were transformed into an electric signal by a mechanical-electric transducer. Signals were amplified and recorded with a signal process system (BL-420 S, Chengdu, China). The neurotoxicity of a sample was evaluated based on its effect on the indirect electrical stimulation-elicited twitch responses of the diaphragm muscle.

The detection of the possible effects on sodium, potassium and calcium ion channels in rat dorsal root ganglion (DRG) neurons with whole-cell patch-clamp technique was performed according to the methods described previously [8,27,28]. DRG cells were acutely dissected from 30-day-old Sprague-Dawley rats of either gender and then maintained in short-term primary culture prior to being used for the experiments. Patch pipettes were made of borosilicate glass capillary tubes and had resistances between 2.0 and 3.0 MΩ. The ion channel currents in experimental DRG cells were recorded at room temperature (20–25 °C). Whole-cell patch-clamp recordings were performed with an Axon 700B patch-clamp amplifier (Axon Instruments, Irvine, CA, USA). The P/4 protocol was used to subtract linear capacitive and leakage currents. Experimental data were acquired and analyzed using the programs Clampfit 10.0 (Axon Instruments, Irvine, CA, USA) and Sigmaplot 12 (Sigma, St. Louis, MO, USA).

In order to investigate the possible effects on the ion channels in insect dorsal unpaired median (DUM) neurons, DUM neurons were isolated from adult cockroaches (*P. americana*) and whole-cell patch-clamp analysis was performed based on the methods described [28,29,30,31]. DUM neurons were enzymatically and mechanically isolated from the terminal abdominal ganglia of the cockroach. Briefly, after the cockroaches were desheathed, their abdominal ganglia were dissected and incubated in the insect physiological solution containing 1 mg/mL protease for an appropriate period of time, followed by neuron dissociation with a thin silver needle. The separated neurons were inspected with a microscope and only the cells that are bright under phase contrast were utilized.

### 4.7. Antibacterial Activity Detection

The bacterial strains used for antibacterial sensitivity testing included *Escherichia coli* (*E. coli*), *Staphyloccocus aureus* (*S. aureus*), *Bacillus subtilis* (*B. subtilis*), *Salmonella typhimurium* (*S. typhimurium*), and *pseudomonas aeruginosa* (*P. aeruginosa*), which were obtained from China General Microbiological Culture Collection Center. The antibacterial activity of the purified peptide against the five bacterial strains was detected using the agar-disc diffusion assay [32]. Bacteria were cultured in LB medium to the exponential phase (OD_600_ = 0.5). 100 µL of each suspension bacterial strain were spread on the agar plate in 35-mm culture dishes. Several Whatman filter paper discs with 6 mm in diameter, after sterilization, were placed on the surface of each medium plate with appropriate spacing. 10 µL of the peptide samples at 50 µM (1.8 µg/disc) were separately applied onto the filer paper discs, and the plates were incubated at 37 °C for 24 h in an aerobic environment to observe the zone of inhibition. Ampicillin (10 µg/ paper disc) was used as a positive control. Antibacterial activity was evaluated by measuring the diameters of inhibition zone in millimeters using a scale. All experiments were performed at least in triplicates.

All studies with laboratory animals were conducted in accordance with the National Research Council’s “Guide for the Care and Use of Laboratory Animals” and applicable institutional national law.

## 5. Conclusions

In the present study, we have purified from the eggs of black widow spiders and preliminarily characterized two novel active proteinaceous components, named Latroeggtoxin-III and Latroeggtoxin-IV, respectively. The biological characterization demonstrated that Latroeggtoxin-III is an insect-specific neurotoxin, without significant effect on mammal animals. Latroeggtoxin-IV shows broad-spectrum anti-bacterial activity and might have an intra-molecular cyclic structure, the determination of which is in progress using a combination of tandem mass spectrometry and partial enzymolysis followed by Edman degradation. This work not only provides new clues for further revealing the toxicity mechanism of black widow spider eggs and expands the understanding of spider toxins, but also supplies new candidates for the development of new insecticides and antibacterial agents.

## Supplementary Materials

Supplementary materials can be accessed at: http://www.mdpi.com/2072-6651/7/3/0886/s1.
