# Supplementary material for: Isolation and Preliminary Characterization of Proteinaceous Toxins with Insecticidal and Antibacterial Activities from Black Widow Spider (L. tredecimguttatus) Eggs
Source: Toxins (Basel). 2015 Mar 16;7(3):886–99. doi: 10.3390/toxins7030886 (PMC4379531; doi:10.3390/toxins7030886)
Supplement: Supplementary file 1 [file toxins-07-00886-s001.pdf]

Supplementary Information

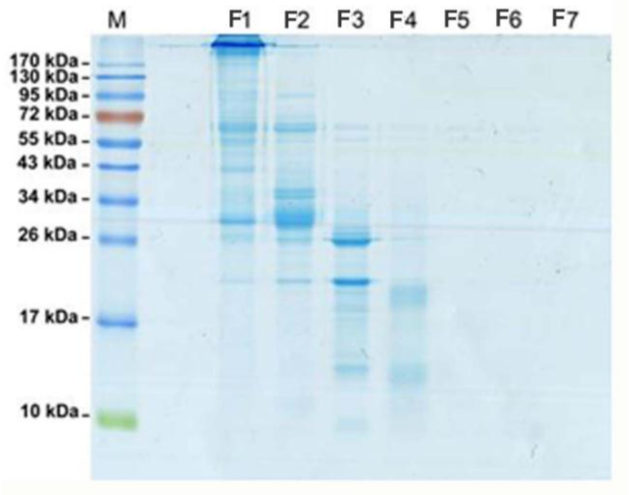

Figure S1. SDS-PAGE image of fractions F1–F7 from molecular sieve chromatography of the extract of black widow spider eggs (Adapted from [10] with minor modification). M, molecular mass marker.

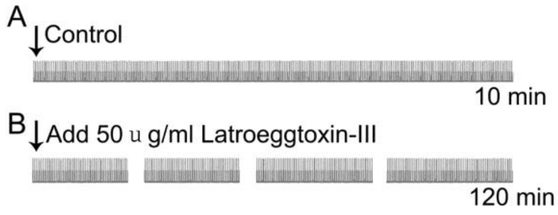

Figure S2. Effect of Latroeggtoxin-III on the neuromuscular transmission in mouse isolated phrenic nerve-hemidiaphragm preparations. (**A**) In control experiment with the preparation immersed in Tyrode's solution without adding Latroeggtoxin-III, there were no significant changes in the amplitude of diaphragm muscle contraction provoked by applying electrical stimulation at the phrenic nerve within at least 2 h. A shows a representative part of the contraction trace. (**B**) The contraction trace of diaphragm muscle after Latroeggtoxin-III was added into the Tyrode's solution at a final concentration of 50 μg/mL. The arrows indicated the start points.

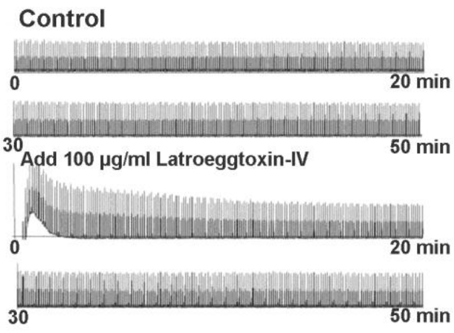

Figure S3. Effect of Latroeggtoxin-IV on the neuromuscular transmission in mouse isolated phrenic nerve-hemidiaphragm preparations.

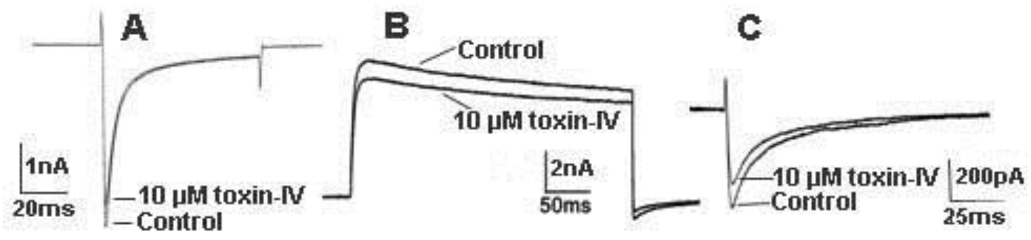

Figure S4. Effects of Latroeggtoxin-IV on voltage-gated sodium (**A**); potassium (**B**) and calcium (**C**) ion channel currents in rat DRG neurons.

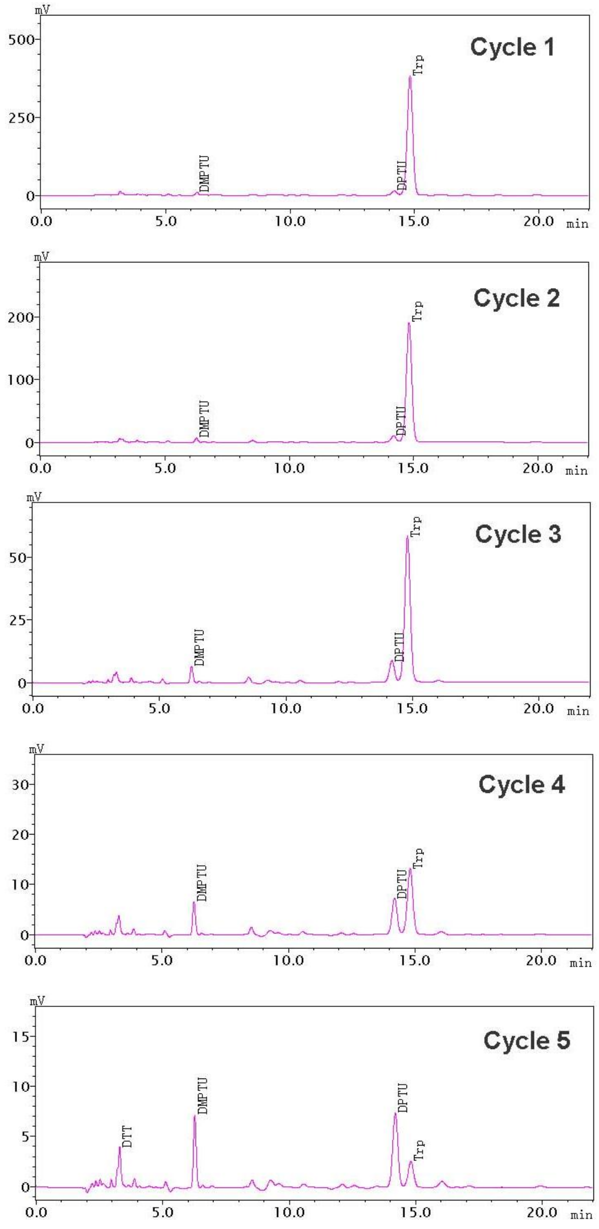

Figure S5. Chromatograms of PTH-AAs at cycles 1 to 5 on sequencing of Latroeggtoxin-IV.
